# Physiology and applied sciences in Nepal: 1st annual conference

**DOI:** 10.1186/2046-7648-3-5

**Published:** 2014-03-01

**Authors:** Ghan Bahadur Thapa, Narayan Bahadur Mahotra, Matiram Pun

**Affiliations:** 1 Mountain Medicine Society of Nepal (MMSN), C/O Nepal International Clinic (NIC), Lal Durbar Marg, GPO BOX 3596, Kathmandu, Nepal; 2 Nepal International Clinic (NIC), Kathmandu, Nepal; 3 Department of Clinical Physiology, Maharajgunj Medical Campus, Institute of Medicine (IOM), TU, Kathmandu, Nepal

**Keywords:** ACPAS, Nepalese physiologists, Nepalese Physiological Society

## Abstract

With the increasing number of medical schools in Nepal, there is an expected increase in the number of Nepalese physiologists. The first medical school was established in the 1970s. We report here about the first annual conference of Nepalese physiologists on 27-28 September 2013 organized by the Department of Clinical Physiology of the Nepalese Army Institute of Health Sciences (NAIHS) and Kathmandu University School of Medical Sciences (KUMS). Nepalese physiologists are trying to form their own physiological society. In this regard, NAIHS and KUMS have played an important role to bring physiologists from different parts of Nepal involved in teaching, learning, and research activities in medical schools. There were a number of foreign invitees (India, Israel, Italy, Japan, and Sweden). There were plenary presentations on the topics that are relevant in Nepal, e.g., high-altitude physiology and wilderness medicine. The final session of the conference was an open session meeting of Nepalese physiologists. There was an open interaction about establishing Nepalese Physiological Society. After much deliberation, there was an agreement to register the society in Kathmandu with the current *ad hoc* committee which will elect the first executive body of the society.

## Background

The first Annual Conference of Physiology and Applied Sciences (ACPAS) of Nepalese physiologists was held on 27 and 28 September 2013 in Kathmandu, Nepal. The 2-day-long conference was jointly organized by the Department of Clinical Physiology of the Nepalese Army Institute of Health Sciences (NAIHS) and the Kathmandu University School of Medical Sciences (KUSMS). The society for Nepalese physiologists is still in conceptual framework. With an increasing number of medical schools in Nepal, there is the need of physiologists. Hence, a physiological society, physiological conference, and collaboration with international scientists seem to be the next steps of Nepalese physiologists. Nepal has long been a proven ground for the hypoxia researches, i.e., physiological and clinical researches at high altitude of the Himalayas among trekkers and Sherpas since more than half a century ago. The conference is thought to be a landmark step in that direction. There were about 200 participants including medical students, doctors, physiologists, and biomedical scientists participating in this conference. The conference provided a useful forum to present their researches, speak about their expertise, and express their opinions regarding future direction of the society. Essentially, it reflects the baseline physiology of Nepalese physiologists and the proposed society, ‘Nepalese Physiological Society (NPS)’. There were a few invited foreign physiologists as well (India, Israel, Italy, Japan, Nepal, and Sweden). The American Physiological Society (APS) provided encouragement and token of incentives for this conference.

## First Annual Conference of Physiology and Applied Sciences (ACPAS 2013)

Day 1: 27 September 2013, Friday

Venue: Army Officers Club, Nepal Army Headquarters, Kathmandu

With the registration and the distribution of the registration kit, the formal program started by welcoming the chief guest Professor Dr. Ram Kantha Makaju Shrestha (vice chancellor of Kathmandu University) and other dignitaries. Professor Dr. Tara Man Amatya, the chairman of ACPAS 2013, delivered the welcome speech. Dr. Amatya mentioned that this conference is a historic event and highlighted the origin of physiology from the very beginning of ancient Ayurveda and Yoga. Dr. Amatya highlighted that the aims of this conference were to discuss scientific advances in physiology, to develop new relationship between different scientists, and to stimulate young scientists and medical students in the field of research. The chief guest (Dr. Shrestha), in his key note to the audience, highlighted the medical situation 20 years ago in Nepal, the emerging medical schools, and the progress Nepal has made so far in this field. Prof. Shrestha further added that education without research is incomplete and gave emphasis on the need for creating more platforms for young scientists.

After the tea break, *Symposium 1: High Altitude Physiology* was chaired by Prof. Dr. Prakash Raj Pande (NAIHS) and Dr. Binaya SJB Rana (Head, Department of Clinical Physiology, IOM, TU, Nepal). Dr. Latika Mohan (All India Institute of Medical Sciences (AIIMS), Uttarakhand, India) presented on ‘strategies for enhancing performance at high altitude’. She elaborated the physiological effects of acute high-altitude exposure and described the measures to enhance performance on entry to high altitude with proper acclimatization, drug uses, and dietary supplementation. Then, Dr. Annalisa Cogo from Italy (Biomedical Sport Studies Center, University of Ferrara, Ferrara, Italy) discussed ‘lung adaptation at high altitude’, highlighting altitude lung physiology, hypoxic ventilatory response (HVR), hypoxic pulmonary vasoconstriction (HPV), and the maladaptive response represented by high-altitude pulmonary edema (HAPE). She added that the extent and the time course of these changes are variable probably due to the individual susceptibility and the ascent profile (acclimatization). This was followed by Dr. Amatya who presented on the topic ‘crisis management in acute mountain sickness’ and focused this issue in the Nepalese context. Dr. Amatya reviewed acute high-altitude illnesses, AMS, HAPE, and HACE and shared a video documentary entitled in Nepali ‘Uchaima (at high altitude)’. Next, Mr. Bhola Raj Pokhrel (KUSMS) from his studies talked about the adaptation in high-altitude natives. In a non-high-altitude-related topic, Professor Yasuo Sakuma (University of Tokyo Health Sciences, Japan) discussed about sexual dimorphism and neural recruitment from animal studies especially in the rat preoptic area. He emphasized steroid-dependent organization of neurons although there have been some latest suggestions that other lipid mediators may play a role, e.g., prostaglandins.

After a short break, *Symposium 2: Neuroscience* started with Prof. Silvert Lindstrom (Linkoping University, Sweden). Prof. Lindstrom discussed about the neuronal control of the urinary bladder in relation to clinical problems, e.g., spinal cord injury. He elegantly reviewed literatures and presented a case on electrical stimulation of bladder mechanoreceptor afferents via an intravesical electrode for the long-term potentiation (LTP) of micturition reflex to restore normal bladder function. This is encouraging and many patients including spinal cord injury ones may benefit from it. Dr. Pushpa Sharma (KUSMS) presented on the neurophysiology of depression. Dr. Sharma described the multifactorial causation of depression and said that depression is influenced more by social factors than biological factors. Dr. Sunil Dhungel (NAIHS) presented data from a transgenic animal model of social and sexual behavior. Dr. Dhungel discussed roles of various neuropeptides in the brain for olfactory preference and sexual behavior. The model and data look convincing although it is likely that there are many mechanisms involved in such complex animal behavior.

*Symposium 3: Cardiopulmonary Physiology* started after lunch break. Dr. Yoshihiro Ishikawa (Yokohama City University, Japan) in his elegant presentation described the role of sympathetic regulation in the physiology of the heart. He highlighted the use of Forskolin, an extract of the plant *Coleus forkohlii*, in treating acute heart failure in Japan. He added that the ancient wisdom in Ayurveda in using this plant in heart disease was made by modifying the side chain and by making it a heart-specific medicine.

To give a clinical perspective to the talks, Dr. Bhagawan Koirala (Director, TUTH, Kathmandu) highlighted the burden of heart diseases in Nepal under ‘preventive cardiology’. Dr. Koirala emphasized ‘what everyone should know?’ about heart disease. He briefly went through a history of cardiac care in Nepal especially open heart surgery that was started in 1997. Dr. Koirala highlighted that every year 17 million people die of heart attack globally. Of all cardiovascular diseases, 80% occur in the developing world. Dr. Koirala mentioned that 20% to 25% of Nepalese (from all parts of the country, urban and rural) have hypertension. Finally, he emphasized healthy lifestyle, diabetes control, and need of more knowledgeable manpower and cardiac rehabilitation program throughout the country. Mr. Pravesh Pokhrial (ADInstruments Inc., India) highlighted the role of technology especially data acquisition systems they have developed in making science and experiments accurate, repeatable, and easier.

The final session of the day was *Oral Presentations by Young Scientists* from various institutions. This session covered a few important topics especially electroencephalogram (EEG) monitoring (Dr. Dev Shah, CMC; Dr. Binu Shrestha and Dr. Rita Khadka) and cardiorespiratory fitness (Dr. Karishma Rajbhandari Pandey, BP Koirala Institute of Health Sciences (BPKIHS) and Ms. Swatika Hada, KUSMS). The first day ended with a cocktail fellowship dinner at Nepalese Army Officers Club.

Day 2: 28 September 2013, Saturday

Venue: Kathmandu University School of Medical Sciences (KUSMS), Dhulikhel, Kavre

The second and final day of the conference started in the auditorium of Kathmandu University School of Medical Sciences, at the outskirt of Kathmandu valley, with a plenary lecture by Prof. K K Deepak (AIIMS, India) on ‘expanding horizons of medical education: empowerment of healthcare professionals’. Dr. Deepak emphasized the enhancement of organizational effectiveness in academic institutions to improve healthcare delivery. He shared some of the policies adopted at AIIMS to improve medical education. Dr. Deepak discussed about faculty development, capacity building of the staff, and pedagogic development as key factors for an institution to succeed, while conflict management, financial administration, professionalism, and ethics also remain important. Healthy communication among the staff within a department and with other departments including hierarchy was important, he said, as new physiology departments are coming up in those upcoming medical institutions.

*High Tea Poster Session* followed with posters in various interesting topics. There were wilderness and environmental medicine topics, e.g., multiple hornet stings and acute kidney injury: a case series (Mr. Yashad Dongol, KIST) and study of pulmonary functions among traffic police in Kathmandu valley (Mr. Hari Sundar Shrestha, KUSMS). The anaphylactic shock and toxins of hornet sting have not been reported well enough in the literature, and the case series will likely fill the gap if followed up for long term. In addition, the pollution of Kathmandu valley is clearly detrimental to the health of traffic police. It should be worrying for the general public as well. There were also new presentations about brain wave activity (EEG monitoring) in a healthy human model (alpha band in EEG in medical students), physiological perturbation (alternate nostril breathing), and disease conditions by the team of Prof. BH Paudel and Dr. Rita Khadka (BPKIHS, Dharan). The researches in Nepalese perspective about human performance, sports, and cardiovascular functions were presented by the teams of KUSMS (Ms. Manisha Bade, Ms. Isabela Thapa, Dr. Biju Shrestha, and Dr. Reena Jha). The researches on cardiopulmonary function in relation to anthropometry, heart rate variability in diabetics, and autonomic functions in sedentary, yogic and athletes highlight the interest of Nepalese physiologists in integrative research.

The brainstem auditory evoked potential (BAEP) responses among hyperthyroidism and hypothyroidism patients (Mr. Vikash Gautam, BPKIHS) are particularly important since there may be an increased incidence of subclinical hypothyroidism in the Nepalese population. Mrs. Puja Paudel (PAHS) had a poster about the role of transient receptor potential cation channel, subfamily v, member 4 (TrPV4) in the rat brain medial preoptic nucleus during acute cold exposure to see the effects on brown adipose tissue.

*Symposium 4: Sports, Yoga and Lifestyle* started with ‘sports, yoga and lifestyle’ by Mr. Kiran Shankar Yogacharya (KU) who discussed benefits of Yoga. He mentioned ancient Yoga methods (physical exercises, breathing exercises, and mental exercises/meditation). Mr. Yogacharya emphasized that Yoga bestows the practitioner with holistic health characterized by sound body, mind, inner joy, peace, and harmony. Prof. Tara Man Amatya presented ‘efficacy and tolerability of yoga breathing in patients with chronic obstructive pulmonary disease (COPD)’. COPD patients taking only short-acting β-2 adrenergic blockers (without previous yoga exposure) after adopting a slower and deeper breathing pattern showed a significant improvement in oxygen saturation with no change in minute ventilation. He concluded that the short-term training in yoga is well tolerated and induces favorable respiratory changes in patients with COPD. Then, Dr. Paudel gave a preview on ‘biomedical research in Nepal’. Dr Paudel covered a historical background of medical science studies which started in the 1970s. He mentioned that the *Journal of Nepal Medical Association* (JNMA) was first published in 1963 AD, and clinical and public health journals have now increased to about six dozens of biomedical journals at present, some of which are indexed in the international indexing system. He said it is important to keep in mind that the number of biomedical journals being established and their indexes in various indexing categories are an important part of the overall biomedical research in any country.

Next, *Symposium 5: Physiology and Allied Science* started with Dr. Das presenting ‘effect of occupational exposure of dust on pulmonary function tests’ among workers of carpet factories and saw mills in Nepal. There was significant decline in forced vital capacity (FVC) and the ratio of forced expiratory volume in 1 s (FEV1) to FVC in carpet factory workers. A similar trend was found in saw mill workers, and workers from both groups had various upper and lower respiratory symptoms. Dr. Jayendra Bajracharya (NAIHS) presented a survey about the ‘use of non-standardized review books by medical students’ in Nepal. Of 200 first- and second-year medical students of NAIHS, 93.1% preferred non-standardized (written by non-experts, not peer reviewed, and lacking references) review books. Dr. Bajracharya concluded that the use of non-standardized review books by medical students is a neglected issue in the field of medical education and may carry a potential negative impact on how medical students gain and retain information.

The scientific sessions of the first Nepalese annual conference of physiology and applied sciences concluded with the note ‘a beginning of continuity of the conference’ from Mr. Bhola Raj Pokhrel (KUSMS). After lunch, Nepalese physiologists gathered to discuss about future strategies in giving an organizational shape to the physiological society of Nepal.

## Nepalese Physiological Society (NPS)

After the scientific session of the final day, there was a general session meeting of Nepalese physiologists to review the past activities and formulate future strategies especially about forming a society. There has been active participation of physiologists from BPKIHS especially in the International Union of Physiological Sciences (IUPS). Although there is no officially registered society for Nepalese physiologists at the moment, the group ‘acted as a representative of Nepalese physiologists’. Hence, in this general open meeting, the group was updated on past activities and attempts on forming a society for Nepalese physiologists. They mentioned that the group has drafted a constitution for the proposed society and formed an *ad hoc* committee along with a list of advisors. Most of the advisors are international physiologists. There was discussion on where the society should be based and how it should proceed. It was agreed that the current *ad hoc* committee should carry out preliminary activities to establish the society and elect a first executive body. The participants agreed to register the society in Kathmandu, the capital city of Nepal. It is important to note that the active initiation and contribution of physiologists from BPKIHS, Dharan were highly appreciated in this regard. Similarly, the physiology department of NAIHS played a crucial role for this meeting, organizing this conference and bringing physiologists from all over the country for the first time. Though there was no timeline declared, it is expected that there will be a society formed for the Nepalese physiologists with a first executive body in the next annual conference next year. Almost all the physiologists in Nepal are currently employed in teaching-learning activities in medical schools. This conference highlighted their keen interest in pursuing research in their respective specialization. Therefore, it is expected to see researches and publications from Nepalese physiologists in coming years. Physiologists were concerned about the lack of research budget/grant for the researches, but it was highlighted that there would be plenty of opportunities for the collaborative researches. At the same time, there could be other possibilities that we could do with local resources involving public-private partnership to carry out researches in Nepal. It is just the beginning, but the future of Nepalese physiologists looks bright ahead.

## Abbreviations

ACPAS: Annual Conference of Physiology and Applied Sciences; AD: Anno Domini; AIIMS: All India Institute of Medical Sciences; AMS: Acute mountain sickness; APS: American Physiological Society; BAEP: Brainstem auditory evoked potentials; BPKIHS: BP Koirala Institute of Health Sciences; CMC: Chitwan Medical College; COPD: Chronic obstructive pulmonary disease; EEG: Electroencephalogram; FEV1: Forced expiratory volume in 1 s; FVC: Forced vital capacity; HACE: High-altitude cerebral edema; HAPE: High-altitude pulmonary edema; HPV: Hypoxic pulmonary vasoconstriction; HVR: Hypoxic ventilatory response; IOM: Institute of Medicine; IUPS: International Union of Physiological Sciences; JNMA: Journal of Nepal Medical Association; KIST: Kathmandu Institute of Science and Technology; KU: Kathmandu University; KUSMS: Kathmandu University School of Medical Sciences; NAIHS: Nepalese Army Institute of Health Sciences; NPS: Nepalese Physiological Society; PAHS: Patan Academy of Health Sciences; SJB: Shamsher Jung Bahadur; TrPV4: Transient receptor potential cation channel, subfamily V, member 4; TU: Tribhuvan University; TUTH: Tribhuvan University Teaching Hospital.

## Competing interests

The authors declare that they have no competing interests.

## Authors’ contributions

MP provided the conceptual framework for the meeting report and edited the manuscript for the final version. GBT prepared the first draft of the manuscript. GBT, MP, and NM prepared and edited the manuscript. All authors read and approved the final manuscript.

